# Sleep, Safety and Social Media: A Qualitative Analysis of Online Discussions About Infant Sleep

**DOI:** 10.1111/cch.70371

**Published:** 2026-11-04

**Authors:** Kirsty L. Hodgson, Kayleigh J. Ison, Madison S. Sharp, Sarah Snuggs, Davina Ledermann, Natalie A. Masento, Katie L. Barfoot

**Affiliations:** ^1^ School of Psychology and Clinical Language Sciences University of Reading Reading UK

**Keywords:** behavioural prevention, family health, infant safe sleep practices, online parent forums, psychosocial support

## Abstract

**Objectives:**

Family norms, values, cultural caregiving expectations and social support can affect infant sleep practices. Evidence‐based infant safe sleep recommendations are an important component of strategies to reduce the risk of sudden, unexpected infant death. This study explored how infant sleep practices were discussed across three online parenting platforms and how safe sleep guidance was discussed and represented within these conversations.

**Materials and Methods:**

Qualitative secondary data (119 posts and 525 responses) were purposively extracted from open threads on Reddit, Mumsnet and Netmums using a predefined sleep‐related keyword framework informed by contemporary safe sleep guidance. Inductive thematic analysis was used to identify and develop themes and sub‐themes within the discussions, followed by content and frequency analyses to quantify the occurrence of identified themes, question types and response types.

**Results:**

Key themes included sleep behaviours, sleeping conditions and competing priorities, highlighting tensions between safe sleep recommendations and parents' practical, emotional and contextual caregiving needs. Practical advice and shared experiences were the predominant response types, while expressions of solidarity provided emotional reassurance. References to formal safe sleep guidance were limited, with parents frequently drawing on peer advice and lived experience when discussing infant sleep.

**Conclusions:**

The findings highlight the role of online peer support in providing spaces where parents discuss and interpret infant sleep practices and suggest the importance of understanding how safe sleep recommendations are discussed and contextualized within peer‐led digital environments. This may inform more effective digital health communication that acknowledges lived parenting realities while supporting safe sleep practices and trust in health communication.

## Key Messages

- This study explored discussions of infant sleep practices across three online parenting platforms and examined how safe sleep guidance was discussed and represented within these conversations.
- Inductive thematic analysis identified themes of sleep behaviours, sleeping conditions and competing priorities, while content and frequency analyses demonstrated the prevalence of these patterns and highlighted limited reference to formal safe sleep guidance.
- The findings highlight the importance of engaging with parent‐led forums to support the communication of safe infant sleep recommendations, address the challenges parents face in applying guidance in everyday contexts and foster trust in health communication.

**Abbreviations**
BPS: British Psychological Society
DC: Darling Child
DM: direct message
DS: Darling Son
GPs: general practitioners
HVs: health visitors
LB: Little Boy
mo: month old
NHS: National Health Service
SIDS: sudden infant death syndrome
SUID: sudden unexplained infant death
UK: United Kingdom
wo: week old

## 1 Introduction

Sudden unexplained infant death (SUID) remains a leading cause of postneonatal mortality in high‐income countries (Moon et al. 2022). Each year, 189 babies die from SUID in the United Kingdom (UK), with 91% of deaths occurring during the first 6 months of life and more than 130 involving unsafe sleeping practices (The Lullaby Trust 2025). The Lullaby Trust, a UK charity dedicated to preventing SUID, promotes evidence‐based safer sleep guidance for parents and healthcare professionals. Although its *Safer Sleep for Babies* campaign, introduced in 1991, contributed to an estimated 80% reduction in SUID initially, unexplained infant deaths in England and Wales have remained relatively unchanged over the past decade, with a rate of 0.28 per 1000 live births reported in 2022 (Office for National Statistics 2024). This plateau suggests that increasing awareness alone may be insufficient to improve safer sleep practices and highlights the need to understand how parents interpret, negotiate and apply guidance within everyday family life.

Infant sleep practices are shaped by dynamic interactions between infant development, parental decision‐making and the wider social environment. Approximately 60% of SUID cases involve a shared sleep surface and 76% involve multiple unsafe sleep practices (Erck Lambert, Shapiro‐Mendoza, et al. 2024). These commonly include soft bedding or pillows, sleeping in adult beds, sofas or chairs and situations where caregiver fatigue, alcohol or drug use increase risk (Erck Lambert, Parks, et al. 2024; Joyner et al. 2010). Beyond the home environment, SUID also occurs in non‐routine settings, such as when infants are cared for by relatives, friends or babysitters, where safe sleep recommendations are less consistently followed (Colvin et al. 2014; Joyner et al. 2010). These findings demonstrate that safer sleep behaviours may be affected not only by knowledge of recommendations but also by changing social contexts, caregiving circumstances and everyday parenting decisions.

Families living in socioeconomically disadvantaged circumstances experience disproportionately higher rates of SUID. Evidence from Scotland demonstrated that declines in sudden infant death syndrome (SIDS) following The Lullaby Trust campaign occurred substantially later in deprived communities compared with more affluent populations (Wood et al. 2012). A systematic review similarly reported a graded association between deprivation and SUID that persisted after adjustment for smoking, birthweight and infant sleep position (Spencer and Logan 2004). Behavioural factors, including maternal smoking and unsafe co‐sleeping practices, account for a considerable proportion of this disparity (Bamber et al. 2016). These findings indicate that wider social circumstances may affect not only exposure to risk, but also the circumstances and opportunities in which families access and implement safer sleep guidance.

Healthcare professionals, including health visitors, midwives and general practitioners (GPs), play an important role in promoting safer sleep guidance. However, translating evidence‐based recommendations into everyday parenting practice is not always straightforward. Co‐sleeping represents one of the most contested aspects of infant sleep guidance. In this paper, co‐sleeping is used as a broad term encompassing arrangements in which an infant sleeps alongside or in close proximity to a caregiver, whereas ‘bed‐sharing’ refers specifically to an infant and caregiver sharing the same sleep surface. While mainstream recommendations advise against routine bed‐sharing because of its association with sleep‐related infant deaths, organizations such as The Lullaby Trust and the NHS also provide harm‐reduction advice on safer co‐sleeping when parents choose or inadvertently fall asleep with their infant (The Lullaby Trust 2024; NHS 2025). Consequently, parents often navigate competing forms of knowledge, balancing formal health recommendations with practical realities, cultural norms and lived experience. Many healthcare professionals continue to discourage co‐sleeping (Altfeld et al. 2017; Kruse et al. 2024), and some parents report concerns about judgement or being perceived as irresponsible when discussing their sleep practices. Emerging evidence suggests that the relationship between bed‐sharing, breastfeeding and infant sleep‐related risk is complex, with outcomes varying according to the presence of recognized risk factors and context (Huber et al. 2024; Moon, Carlin and Hand 2022; Tappin et al. 2023). This may contribute to uncertainty for parents and highlights how infant sleep decisions can involve negotiation between formal guidance and experiential knowledge (Altfeld et al. 2017; Pease et al. 2021; Kruse et al. 2024).

Although adherence to safer sleep guidance is fundamental to reducing SUID risk, implementing recommendations such as Basis Baby Sleep Information Source (2022) within family life remains challenging. Maruyama and Hageman (2022) emphasized the importance of consistent health messaging for new parents. Cole et al. (2022) found that although awareness of safer sleep guidance was high, practical challenges, cultural beliefs, conflicting advice and previous experience frequently limited implementation. Likewise, Pease et al. (2017, 2021) demonstrated that many parents prioritized infant comfort, maternal instinct and previous experience when making sleep‐related decisions, despite understanding formal recommendations. These findings suggest that parental decision‐making may be affected not only by health guidance but also by social norms, emotional reassurance and lived experience. When recommendations appear difficult to apply or conflict with everyday parenting realities, parents may turn to alternative sources of support and information to help them navigate these challenges.

Parenthood represents a major developmental transition characterized by new responsibilities, uncertainty and substantial informational and emotional needs (Maylott et al. 2023). Psychosocial support plays an important role during this period. Kruse et al. (2024) argued that parental decision‐making around infant sleep reflects a complex interaction between lived experience and socio‐cultural expectations. Increasingly, support has shifted from face‐to‐face networks towards digital communities, including discussion forums, blogs and social media (Mohd Roffeei et al. 2015). Parents use online communities to seek advice, compare experiences and obtain emotional support regarding infant care, including sleep (Plantin and Daneback 2009). Online parenting platforms can represent more than sources of information; they may also provide interactive spaces in which parents seek reassurance, exchange experiences and information and develop shared understandings of infant care (Plantin and Daneback 2009; Thoren et al. 2013; Francisco 2024).

Digital engagement has increased substantially over the past decade, with the COVID‐19 pandemic further accelerating the use of online communication technologies (Ammar et al. 2020). Research has also demonstrated that parental media use can affect broader family functioning and child development (Paoletti et al. 2024). Holopainen et al. (2025) found that higher levels of general social support were associated with fewer parental burnout symptoms during the COVID‐19 pandemic. In contrast, Thoren et al. (2013) examined support and information sharing within Facebook communities for parents of preterm infants primarily centred on interpersonal support and information sharing. Similarly, Francisco (2024) analysed 796 Reddit threads concerning parenting support and identified discussions relating to child health and development, childcare, self‐identity and the prenatal and postpartum periods. Mothers sought both emotional and informational support across the subreddits examined. These studies suggest that online parenting communities have become important environments in which health information, social norms and lived experience intersect. Although these platforms may provide valuable psychosocial support, they may also reinforce conflicting interpretations of health guidance or disseminate inaccurate information. Understanding how psychosocial support and information sharing occur in digital contexts may provide useful insights for improving health communication (Kanchan and Gaidhane 2023; Bennett et al. 2024).

### 1.1 The Present Study

Despite the growing reliance on online parenting communities, relatively little is known about how infant sleep practices are discussed within these spaces, what information parents seek or how safe sleep guidance is discussed and represented. Addressing this gap may provide valuable insights into how parents interpret and negotiate infant sleep guidance in everyday practice.

The primary aim of this study was to identify the principal themes relating to infant sleep practices discussed across three online parenting platforms using inductive thematic analysis. The secondary aim was to examine how safe sleep guidance (The Lullaby Trust 2024) was discussed and represented within these conversations.

## 2 Methods

### 2.1 Design

This study employed a naturalistic qualitative design using naturally occurring discussions posted on publicly accessible online parenting platforms. Unlike interview‐ or survey‐based research, the study analysed discussions that had been generated independently of the research, without researcher intervention, enabling exploration of parents' naturally occurring discussions surrounding infant sleep practices. Reddit, Mumsnet and Netmums were purposively selected because they are well‐established online parenting communities that host active discussions relating to infant care and sleep. Although the platforms differ in their format and user communities, together they enabled exploration of naturally occurring discussions across multiple digital parenting environments.

### 2.2 Ethical Considerations

The study carefully considered ethical issues, and the authors affirmed that the ethical principles were followed. This study adhered to the British Psychological Society (BPS) Ethics Guidelines for Internet‐Mediated Research (BPS 2021b) and the BPS Code of Ethics and Conduct (BPS 2021a). Ethical approval was obtained from the University of Reading. Following ethical approval, social media groups and site administrators were initially contacted prior to extracting user responses from pages to explain the research aims and request permission to access the group data (where required). As this study utilized secondary data that had already been published online, it was not possible to obtain informed consent from all users of each social media group. To overcome this, a post was published on the main page of the group by the researcher to all members that explained the aims and rationale of the research, data protection information and assurances of the anonymization of identifying information during extraction and analysis (no profile details, user handles or identifiable information were retained). Details were also provided to inform individuals on how to ‘dissent consent’ (not consent) and subsequently withdraw their data from use in the research, which they could do by commenting on the researcher's post or by emailing the researcher. This research did not require proactive input from participants, unless they chose to withdraw their data from inclusion in the study by commenting on the post or emailing the researcher at any time. None of the participants expressed concerns or requested data extraction throughout or after this study. Nevertheless, we acknowledge that owing to the asynchronous nature of online communities, not all users whose posts were included may have viewed the study notification. Although this limits the extent to which awareness of the dissent consent process can be assumed, the study adhered to BPS guidance for internet‐mediated research (BPS 2021b) using publicly accessible data, anonymization procedures and provision of opportunities for participants to withdraw their data. This also follows guidance from the Information Commissioner's Office, which stipulates that if personal data are obtained from publicly accessible sources (such as social media), individuals must be provided with private information (Information Commissioner's Office 2023).

### 2.3 Platform Strategy and Data Extraction

Publicly accessible parent‐focused online communities were purposively selected to examine naturally occurring discussions relating to infant sleep and safe sleep practices. These platforms enabled exploration of how parents sought, shared and represented information within digital parenting communities. Consistent with a constructivist perspective, the study recognized that knowledge is co‐constructed through social interaction and shared experience within online communities (Alismaiel et al. 2022).

Data extraction was undertaken between July and August 2022. To capture contemporary discussions while maintaining a manageable dataset, only posts published within the preceding 6 months were eligible for inclusion. A predefined cut‐off point was established prior to data collection to allow sufficient time for data extraction and preliminary analysis. Therefore, data were extracted across 6 weeks, and posts were eligible for extraction if they were posted with a date of less than 6 months. Posts were included if they contained the keyword ‘sleep’ or associated synonyms. The search strategy was developed a priori using contemporary evidence‐based infant safe sleep recommendations published by the American Academy of Pediatrics, the National Institute for Health and Care Excellence (NICE 2021), The Lullaby Trust (2022) and the Basis Baby Sleep Information Source (Moon et al. 2022). Keywords reflected the principal domains addressed within contemporary safe sleep guidance and included both clinical terminology and commonly used parental language. The complete keyword framework, including all search terms, spelling variants and synonyms used during data extraction, is provided in Supplementary File 1. The predefined keyword framework was used solely to identify eligible discussions and did not constitute a coding framework for analysis.

Posts were eligible for inclusion if they (i) were publicly accessible, (ii) were posted within the predefined sampling period, (iii) related to infant sleep or infant safe sleep practices and (iv) contained one or more terms from the predefined keyword framework. Posts were excluded if they related primarily to older children or adult sleep; were duplicate discussions, moderator announcements or administrative posts; were unrelated to infant sleep or safe sleep practices; or were inaccessible owing to privacy restrictions. As searches were conducted manually across platforms with differing search functions, a complete numerical record of all initially screened posts was not retained. Eligibility was determined through sequential screening against the predefined inclusion and exclusion criteria, resulting in a final dataset of 119 eligible posts.

Specific parent‐focused national and regional social media platforms were identified. Reddit, Mumsnet and Netmums were selected purposively because they represent distinct but established online parenting communities with differing user demographics, discussion formats and patterns of engagement. Sampling across multiple platforms was intended to capture variation in naturally occurring discussions relating to infant sleep rather than to achieve proportional representation of individual platforms. Forums that mentioned the word ‘sleep’ were utilized for searches. Eligible posts were copied verbatim into Microsoft Excel for organization and analysis. The original post and the first 10 direct responses were extracted from each discussion thread. This sampling strategy was determined a priori to ensure consistency across threads of varying length while retaining the initial exchanges that most directly addressed the original post. Replies to subsequent comments were not extracted.

### 2.4 Parenting Media

One hundred nineteen posts and 525 associated responses were selected for extraction. The adequacy of the dataset was considered in terms of information power (Malterud et al. 2016), whereby the richness, relevance and diversity of naturally occurring discussions across three parenting platforms provided sufficient information power to address the study aims. These posts were located in various parenting forums on babies' sleep; the majority were from Mumsnet (*n* = 76) and Reddit (*n* = 42) and one discussion on infant safe sleep on Netmums (*n* = 1). Regarding data protection, all data were taken from online websites where parents had willingly posted on different forums to obtain advice from others.

### 2.5 Coding and Analysis

The qualitative data were analysed using thematic analysis to identify and develop recurring patterns and themes within parents' discussions of infant sleep. Following development of the thematic structure, content and frequency analyses were used to quantify the occurrence of the identified themes, sub‐themes, post types, question types and response types. Therefore, thematic analysis was used to identify and interpret patterns within the data, while content and frequency analyses were subsequently used to describe the prevalence of these patterns across the dataset.

Three researchers contributed to the coding process. All three independently reviewed posts and responses from each platform and generated initial codes inductively from the content and meaning of the data. Thematic analysis followed the principles outlined by Braun and Clarke (2006), including familiarization with the data, generation of initial codes, development and review of candidate themes, defining and naming themes and producing the final analysis. Coding decisions were subsequently compared across researchers, and differences in interpretation were discussed until consensus was reached. Codes were not fixed a priori; they were added, combined, refined or renamed when further review of the dataset indicated that the initial coding did not adequately capture recurring meanings or distinctions within the data. The refined coding structure was then used to guide analysis across the dataset.

The analysis progressed iteratively from raw data extracts to initial codes, meaning codes, sub‐themes and overarching themes. Initial codes captured the substantive topic or issue represented within a post or response. Related codes were then compared across posts and platforms and grouped according to shared meanings. These groups were subsequently refined into sub‐themes and overarching themes through repeated review, comparison and discussion between the three researchers. For example, extracts concerning co‐sleeping, parental exhaustion and difficulties balancing parental and infant needs were initially coded according to their substantive content and subsequently grouped into higher order categories reflecting shared meanings. Illustrative examples of this progression from raw data to initial codes, meaning codes, sub‐themes and overarching themes are provided in Supplementary File 2.

Following development of the thematic structure, content analysis was used to systematically categorize and quantify the identified patterns. Content analysis has been used as both a quantitative and qualitative approach to the systematic examination of textual data (Kolbe and Burnett 1991; Kleinheksel et al. 2020). In the present study, frequency analysis was used to quantify the occurrence of themes, sub‐themes, post types, question types and response types identified through the qualitative analysis. This enabled the qualitative patterns identified through thematic analysis to be examined alongside their prevalence across the dataset and provided the basis for the quantitative summaries presented in Figures 2 and 3 and Table 1.

**TABLE 1:** Guidance sources.

Guidance source	Attitude to source			Platform		Situation		Example quote
	Positive	Negative	Neutral	Mumsnet	Reddit	Post	Post response	
The Lullaby Trust (*n* = 6)	5	0	1	6	0	3	3	‘I've been following all recommendations from the lullaby trust’. (P137) ‘As long as you are following lullaby guidelines it is perfectly safe’. (P137r4) ‘Have you tried safe bed sharing? Lullaby trust explains how it works’. (P290r1)
Google (*n* = 8)	6	1	1	7	1	1	7	‘When I Google I just get unsafe advice about propping her up with rolled towels and blankets to keep her on her back, which I'm obviously not going to follow’. (P275) ‘Lots of techniques to overcome if you Google but tricky to find the one that works for your child’. (P312r2) ‘We are one of the only countries that promotes against bed sharing … they can google it if they care’. (P637r4)
Assigned health professional (*n* = 17)								‘Chat with GP to see if they can help’. (P217r10) ‘If anyone has any tips that do not involve him screaming for hours, I am all ears. I specify that because it's all the health visitors can ever tell me’. (P336) ‘My HV told me it was because I was feeding to sleep and I needed to start putting her down awake. It took weeks to sort’. (P75r2) ‘HV put the fear of God into me about co‐sleeping generally’. (P163r3)
General practitioner/doctor (*n* = 6)	2	2	2	4	2	3	3	
Midwife (*n* = 3)	2	1	0	2	1	1	2	
Health visitor (HV) (*n* = 8)	3	5	0	7	1	3	5	

*Note:* The table summarizes guidance sources mentioned in social media discussions on infant sleep. Attitudes towards sources vary, with most comments positive towards The Lullaby Trust and Google and mixed attitudes towards healthcare professionals. Example quotes illustrate parent reliance and differing trust in guidance sources.

The safe sleep guidance published by The Lullaby Trust (2024) and Basis Baby Sleep Information Source (2022) was not used as a deductive coding framework. Instead, the guidance informed the development of the search strategy and was subsequently used to contextualize and interpret findings relating to infant safe sleep practices. This distinction ensured that the thematic structure was grounded in the naturally occurring discussions within the dataset rather than being imposed by predefined guideline categories, while allowing the findings to be considered in relation to contemporary safe sleep recommendations.

The analysis therefore combined an inductive qualitative examination of naturally occurring discussions with subsequent content and frequency analyses. This approach allowed the study to identify what parents discussed and how these discussions were characterized while also examining the relative prevalence of the resulting themes and discussion patterns across the dataset.

## 3 Results

Thematic focus varied across platforms, as Reddit threads were more conversational and interactive, while Mumsnet featured more advice‐eliciting longer posts. Posts were predominantly support‐ or advice‐seeking, often framed with emotional distress, uncertainty or the challenges of navigating conflicting information from online content or health professionals. Thematic analysis identified the overarching themes and more specific sub‐themes within the 119 posts. Frequency analysis was subsequently used to quantify the number and proportion of posts coded to each overarching theme and specific sub‐theme. As a single post could contain multiple themes and/or sub‐themes, frequencies were calculated independently and were not expected to sum to the total number of posts. The key themes and sub‐themes from the data were Theme 1, competing priorities (Sub‐Themes 1.1, parent vs. baby's needs; 1.2, following safe sleep guidelines; and 1.3, worry); Theme 2, sleep behaviours (Sub‐Themes 2.1, regression; 2.2, feeding to sleep; and 2.3, difficulty separating from the parent); and Theme 3, sleeping conditions (Sub‐Themes 3.1, co‐sleeping; 3.2, temperature; 3.3, sleeping position; and 3.4, sleeping set‐up). These are shown in Figure 1.

**FIGURE 1:**
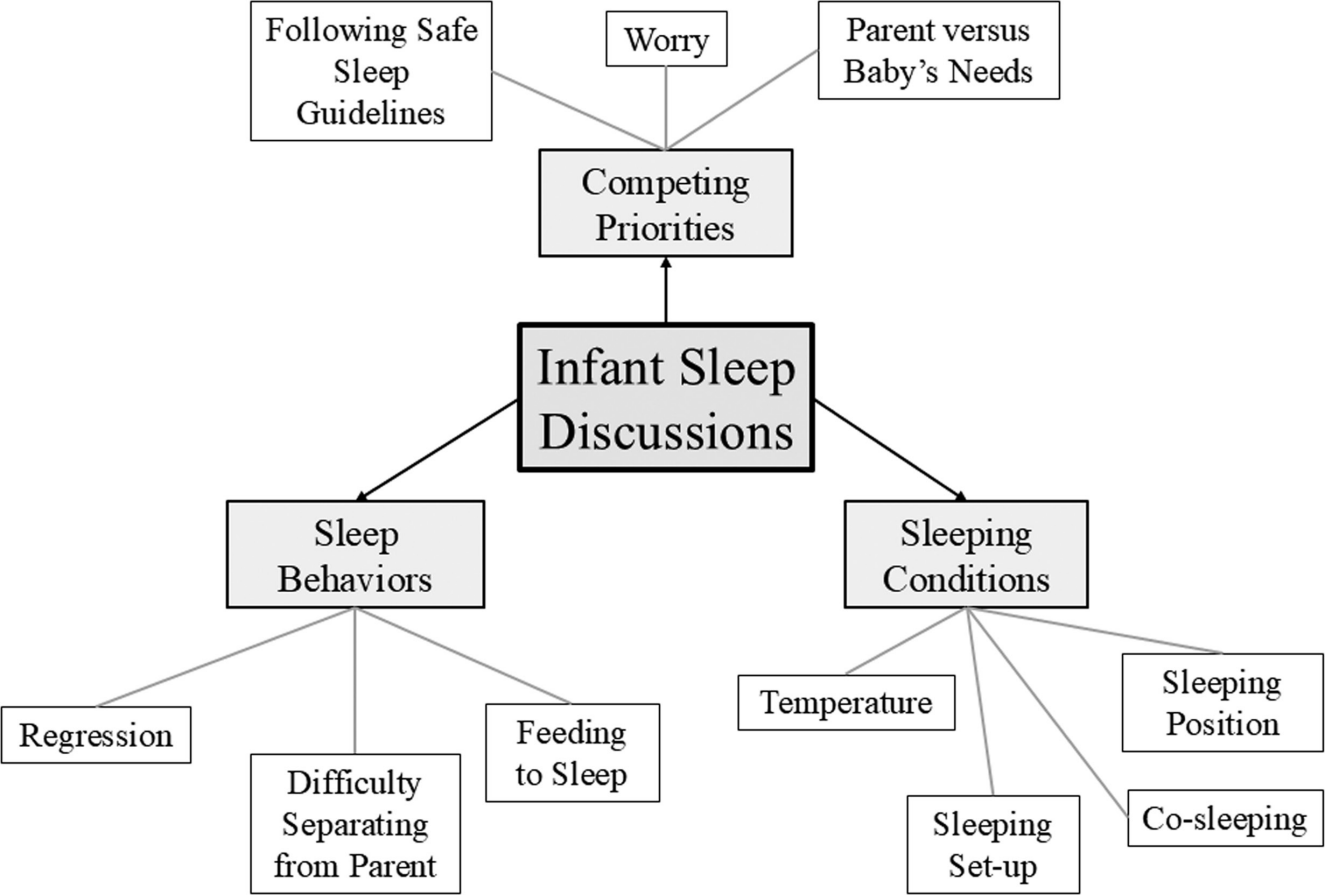
Thematic map of infant sleep discussions. Thematic map illustrating the main themes identified in social media discussions about infant sleep. Key themes include *competing priorities*, *sleep behaviours* and *sleeping conditions*, each with interconnected sub‐themes showing how parents balance infant needs, safety guidance and environmental factors.

### 3.1 Quantitative Content Analysis Results

To address the aim of identifying the proportion of discussion posts related to infant sleep, thematic dominance was examined through quantitative content analysis. Figure 2 presents prevalence‐based theme‐level summaries and sub‐theme‐level summaries, alongside the associated data table.

**FIGURE 2:**
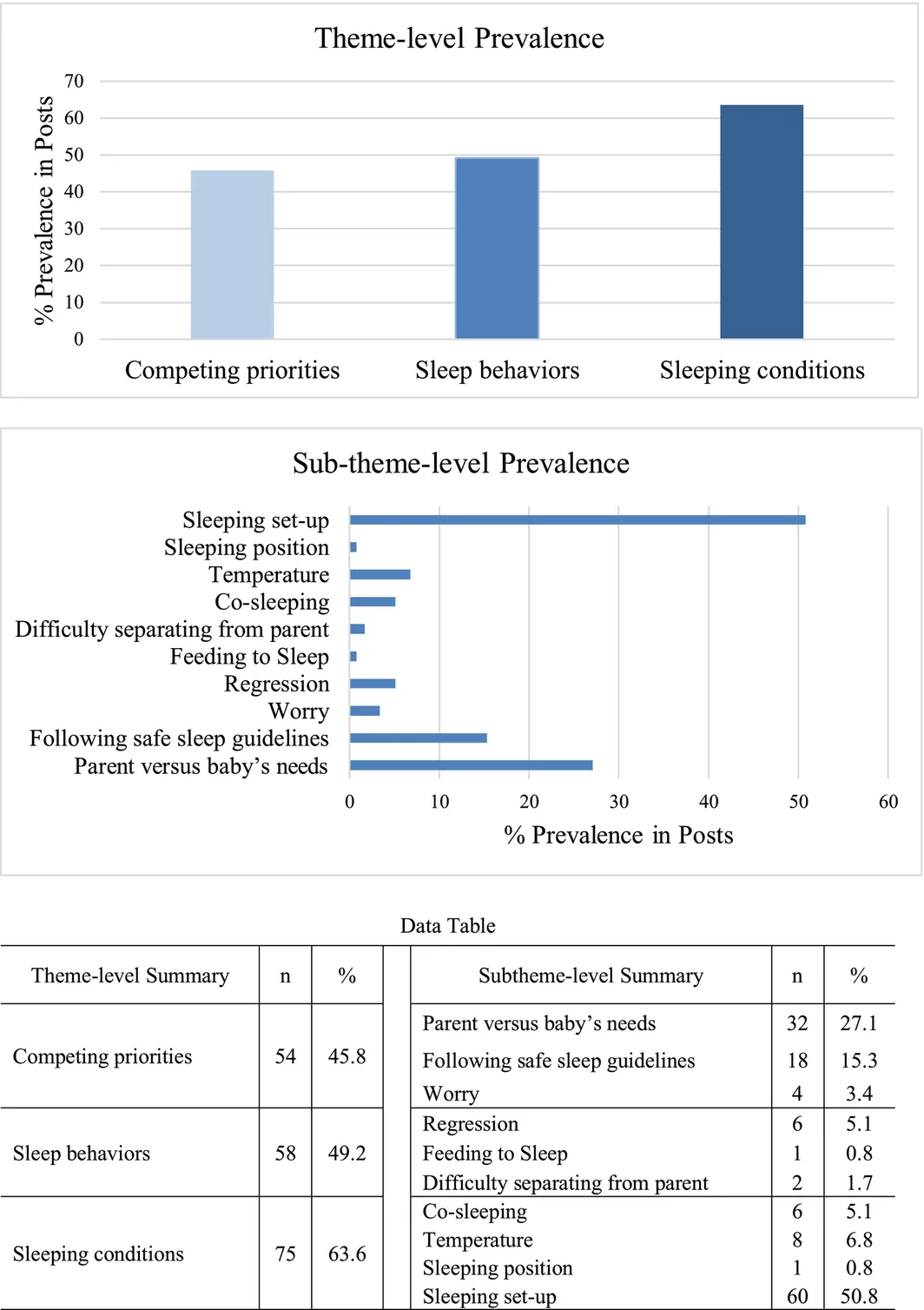
Thematic prevalence. A vertical bar chart showing the main theme‐level prevalence of posts in social media about infant sleep. *Sleeping conditions* was most common, followed by s*leep behaviours* and c*ompeting priorities*. A horizontal bar chart showing the sub‐theme‐level prevalence in social media posts about infant sleep. Sleeping set‐up was the most frequent sub‐theme, followed by *parent* versus *baby's needs* and *following safe sleep guidelines*. Other sub‐themes such as *temperature*, *co‐sleeping*, *regression* and *worry* occurred less often, while *sleeping position*, *feeding to sleep* and *difficulty separating from parent* were least mentioned. An accompanying data table summarizes the thematic prevalence of social media discussions relating to infant sleep practice. Themes include *competing priorities* (45.8%), *sleep behaviours* (49.2%) and *sleeping conditions* (63.6%). Sub‐themes show the proportion of posts mentioning specific issues, such as *parent* versus *baby's needs* (27.1%), *following safe sleep guidelines* (15.3%), *regression* (5.1%), *feeding to sleep* (0.8%), *co‐sleeping* (5.1%), *temperature* (6.8%) and *sleeping set‐up* (50.8%).

Figure 2 summarizes the prevalence of themes identified across the dataset. Sleeping conditions was the most prevalent overarching theme, occurring in 63.6% (*n* = 75) of posts, followed by sleep behaviours (49.2%, *n* = 58) and competing priorities (45.8%, *n* = 54). At the sub‐theme level, sleeping set‐up was the most frequently discussed topic (50.8%, *n* = 60), followed by parent versus baby's needs (27.1%, *n* = 32) and following safe sleep guidelines (15.3%, *n* = 18). Less commonly discussed topics included temperature (6.8%, *n* = 8), co‐sleeping (5.1%, *n* = 6), regression (5.1%, *n* = 6) and worry (3.4%, *n* = 4), while difficulty separating from parent (1.7%, *n* = 2), feeding to sleep (0.8%, *n* = 1) and sleeping position (0.8%, *n* = 3) were the least frequently represented. As individual posts could be coded to more than one overarching theme and/or specific sub‐theme, percentages were not expected to sum to 100%; because many posts contained more than one theme, the percentages exceeded 100%. Thematic analysis explored the thematic focus and meaning of the discussion posts as follows.

### 3.2 Theme 1: Competing Priorities

There were references to competing priorities throughout many of the posts. Three specific sub‐themes were identified within this broader theme: parent versus baby's needs, following safe sleep guidelines and worry.

#### 3.2.1 Sub‐Theme 1.1: Parent Versus Baby's Needs

Many posts showed that there could be difficulty balancing parents' needs and the baby's needs, one example being: ‘every day is a constant battle for sleep and I'm terrified that I'm running on empty and unable to take care of him properly’ (P39). This post indicates that what the child needed to help him sleep had adverse effects on the parent, to the extent that they worried they might be unable to care for their child. Another parent mentioned struggling to be well rested: ‘Takes about an hour between sleeps to get him back down … I'm absolutely knackered and just spend every night so frustrated’ (P11). These highlight that many parents cannot get sufficient sleep as their primary focus is on their child's needs. Numerous parents mentioned difficulty in completing tasks: ‘I'm struggling to do anything with him in my arms all the time’ (P20). The parent was ‘struggling’ to complete activities due to carrying their sleeping child and was seeking advice on how to settle their child in their sleep space (Moses basket). Another parent reported a similar experience: ‘He will also only nap on me and whilst I still love the cuddles, I can't get anything done’ (P16). Despite the child napping on the parent being somewhat enjoyable, it was still a barrier to the parent functioning.

These infant needs also disrupted parents' work and home life: ‘I'm back at work now and struggling as I get no sleep when she's in bed with me’ (P42). The parents in this post were co‐sleeping, but the sleep context made it difficult for one parent to sleep. One parent mentioned that their child's sleeping habits were a barrier to time with their partner: ‘my partner works all day and the only time we would get to see each other is evenings but I'm too busy trying to get LB (Little Boy) to sleep’ (P52). This illustrates that there are a range of familial needs and dynamics that infant sleep can disrupt. Parents described approaches such as allowing the baby to fall asleep in their arms, which some presented as a pragmatic way of settling their child. Although these approaches could help settle the infant, parents also described consequences for their own sleep, relationships, daily activities and work. However, parents posting this information were also seeking ways to change these patterns so that their own needs and those of the family could be better met.

#### 3.2.2 Sub‐Theme 1.2: Following Safe Sleep Guidelines

Parents described trying to follow safe sleep guidance throughout many of the posts; however, some spoke about this intent more explicitly: ‘I've been following all recommendations from the lullaby trust’ (P29). This demonstrated the parents' intention to learn what is safe for their child and to act on it. Another parent also mentioned their adherence to the guidelines: ‘My 3 day old sleeps in her own next‐to‐me‐style cot, in a sleep bag. I have adhered to all of the Lullaby Trust advice for safe sleep’ (P53). Again, this parent explicitly mentioned The Lullaby Trust and that they have followed the advice.

Some parents discussed the guidelines but mentioned how they planned to adapt co‐sleeping recommendations, for example: ‘I know the guidelines are no blanket or a very thin sheet. I cannot sleep without some weight on me so I have always used a regular comforter, but sleep with the edges that would be towards her tucked under me’ (P109). This emphasizes that despite not rigidly adhering to the guidelines, the parent attempted to make sleep safer, while also trying to make it comfortable for both. The context that they are then reaching out online for further guidance and support indicated that although parents valued the guidelines, they also valued the opinions and experiences of other parents. In addition to shared lived experiences and anecdotes of social stories, there were numerous references to other guidance sources. Table 1 provides the frequencies and indicative quotes from the messages, referring to the guidance sources.

There were multiple references to dedicated healthcare professionals, including GPs, HVs and midwives. Attitudes towards the value and credibility of the sources were variable, with health professionals sometimes described as lacking solutions or being unhelpful in some cases. The Lullaby Trust (2024) were referenced explicitly on six occasions, whereas Google was specifically signposted as a guidance source on eight occasions. The discussion themes reflected many of the practical aspects of infant safe sleep addressed within *Safer Sleep for Babies: A Guide for Parents* (The Lullaby Trust 2024), particularly sleeping arrangements, the infant sleep environment and bedding. However, the discussions primarily reflected parents' interpretation and application of these topics in everyday practice rather than explicit references to, or evaluations of, the guidance itself.

#### 3.2.3 Sub‐Theme 1.3: Worry

Throughout many posts, parents also stressed their concern and worry about their child's sleep. Worry was linked to multiple themes and sub‐themes mentioned, such as difficulty separating from parents and getting to sleep: ‘Am I cruel? Is this going to negatively affect her?’ (P54). This emphasizes that despite the parents' best efforts to get their child to sleep and trying multiple methods to achieve this, they remained worried that letting their child cry out was cruel despite this seemingly working. The questions asked also suggested that the parent wished to seek reassurance; another parent said, ‘I haven't slept in two days, waking up every 10mins because I'm so paranoid as I know it's important for babies to sleep on the back’ (P19). Contact napping may be defined as infant sleep that occurs while being held in physical contact rather than in a separate sleep space. A parent who contact napped said: ‘I just worry that she's going to wake up and I won't be there and she'll be so upset’ (P115). This also highlights the difficulty of moving from co‐sleeping to independent sleep surfaces in the same room, which created separation anxiety not only for the child but also for the parent in some posts.

### 3.3 Theme 2: Sleep Behaviours

Many parents shared in posts and comments what they were already doing to support their child's sleep behaviours, an example of this being: ‘We have tried shushing, cuddling, rubbing his back, patting his bum, white noise but nothing works’ (P24). Parents often tried multiple techniques to get their child to sleep before reaching out for help from other parents online. Parents reported various attributions for their children's sleep behaviour reflecting the sub‐themes of regression, feeding to sleep and difficulty in separating from parents.

#### 3.3.1 Sub‐Theme 2.1: Regression

Temporary disruption in sleep patterns associated with developmental milestones, often termed ‘sleep regression’ by parents, can lead to increased night waking, difficulty falling asleep or changes in nap routines, typically lasting a few weeks (Burnham et al. 2002; Bryan et al. 2022). Although common, they can be challenging for both children and caregivers (Škodová et al. 2022). Many parents discussed sleep regression, noting a change from how their child's previous sleep pattern: ‘My previous “good sleeper” is now waking up hourly at night. Any tips on how long this lasts and if things will ever go back to “normal”’ (P4). This highlighted the change in the child's sleep and suggested that the parent wished for their sleep to return to ‘normal’, they sought advice on how long they may have to experience it. Another post said: ‘When he was a newborn he slept in the day (on me) so I could at least rest but now he needs my constant attention and interaction and isn't a good napper’ (P57). This demonstrates that sleep regression affects not only sleep at night but also naps throughout the day, and this change in sleep can make it difficult for parents to abide by their normal routine.

Some parents specifically labelled their infant as being in regression: ‘Pretty sure we in the regression. She screams before every nap and bedtime she wakes a lot in the night’ (P14). Parents stressed how difficult it was to cope with their sleep while they were going through regression. Several parents described sleep regression and struggled with the associated disruption their own sleep quality and daily routines. They reached out on social media during this period after they had exhausted their own options on how to help their child sleep. The theme of sleep regression and the inherent disruption in sleep patterns associated with developmental milestones highlights the importance of considering sleep trajectory patterns at different ages, as challenges can change greatly between different developmental milestones. Therefore, parents found themselves adjusting to a continually evolving ‘new normal’ as their child developed, and some sought support to manage these ongoing transitions in sleep routines.

#### 3.3.2 Sub‐Theme 2.2: Feeding to Sleep

Some parents mentioned feeding their child while the child fell asleep: ‘The only way to get her back to sleep after she wakes is to pick her up and nurse her, or she has more milk and drifts off herself’ (P2), suggesting that the parent had concluded that feeding the child to sleep was the only method of getting the child to sleep that worked for them. Other parents had similar experiences, however, some held more negative views towards it: ‘Have just been feeding back to sleep, knowing that all phases pass, but I'm really getting a bit bored of it’ (P18). Another parent was concerned about nursing to sleep: ‘normally we nurse to sleep for every nap and once when he wakes up in the middle of the night … I've researched and have been told nursing to sleep isn't ideal’ (P97). This indicated that many found that nursing their children helped them to fall asleep; however, some parents wanted to change this routine, so they sought advice online.

#### 3.3.3 Sub‐Theme 2.3: Difficulty Separating From Parent

Some posts reported their difficulties with sleeping independently: ‘The last three nights have been HELL. She just won't sleep unless I'm holding her, period. She screams hysterically when she wakes in her crib’ (P84). Parents described the distress that their infants experienced: ‘won't sleep unless she's laying on one of us. Every time we put her in her crib her eyes pop open and that's it she's awake again and starts getting restless until she cries and we have to pick her up’ (P56). This showed how important contact with the parent was to the child and that the parent believed that the only option to get their child to sleep was through physical comfort.

The term ‘separation anxiety’ was also mentioned by several parents: ‘Since starting nursery he seems to have developed severe separation anxiety especially at night so he no longer goes in his cot’ (P32). This highlighted parents' beliefs about why their child would not sleep and suggested that they were initially fine while sleeping alone but that this can change depending on what is happening in their lives. Parents often reached out for advice on how to support their child to sleep alone at night when there had been a change from their usual sleeping behaviour or when it conflicted with their own needs, as discussed in the competing priorities theme. Although co‐sleeping generated substantial discussion, sleeping conditions overall represented the most prevalent theme identified across the dataset (Figure 2).

### 3.4 Theme 3: Sleeping Conditions

The theme of sleeping conditions reflected a range of different aspects that parents considered when trying to support their children to sleep. This theme included the sub‐themes of co‐sleeping, temperature, sleeping position and sleeping set‐up.

#### 3.4.1 Sub‐Theme 3.1: Co‐Sleeping

Co‐sleeping was discussed, with conflicting views. Some enjoyed co‐sleeping and did not want to give up. One parent stressed how much they appreciated co‐sleeping: ‘So it's been about 6 months now, and we also contact nap on the glider in her nursery during the day. I LOVE sleeping with her and don't feel quite ready to quit bed sharing’ (P98). However, others co‐slept out of what they considered to be necessity; for example, ‘when DS (Darling Son) wakes up in the night we just end up bringing him into our bed to get some sleep because we are just so tired’ (P24).

One parent wanted to begin co‐sleeping: ‘We're very interested in cosleeping now but it feels like there is so much data and personal opinion against it’ (P102). This post acknowledged others' views and highlighted that it could be a controversial topic among parents, particularly when conflicting guidance is presented to new parents. Another parent wished to continue co‐sleeping but to adapt to their current routine: ‘My husband is now joining us in bed after 7 months of sleeping on the sofa. What's the common advice of where to lay our baby down when both parents shares the bed?’ (P108). The parent did not wish to stop co‐sleeping but was aware that it was important to consider alternative sleeping positions to keep their baby safe. Other parents wished to stop co‐sleeping: ‘my 13wo (week old) has gotten used to bedsharing … it's not sustainable for us, and I want to get him back into our bedside sleeper or the crib’ (P12). Despite their need to bedshare initially, they wanted to stop and reached out for advice on an online forum. Parents sought help online for diverse reasons related to co‐sleeping, some wanted to improve their methods and others wanted to stop when possible.

#### 3.4.2 Sub‐Theme 3.2: Temperature

Posts often sought advice and expressed anxiety about their child's temperature: ‘Any tips for keeping baby cool? I feel anxious about the heat’ (P7). They were keen to ensure that their child was the correct temperature: ‘Just wondering how people are dressing their babies for bed on the warm nights we are having in the UK? … I'm constantly checking her neck and tummy and she seems cool enough’ (P26). Although temperature was spoken about slightly less than some other themes, it greatly concerned parents, particularly the idea of their child being too hot at night, suggesting that they may have been aware of associated safe sleep guidance.

#### 3.4.3 Sub‐Theme 3.3: Sleeping Position

Comparable with temperature, another topic that parents were concerned about was their child's sleeping position, with most parents being aware of sleep guidance. Three posts were coded to this sub‐theme. One parent described repeatedly trying to position their infant on their back:

I try putting them down on their back every night‐time feed and nappy check, but within seconds they are on their side … I know the rule is to put babies to sleep on their backs but the only way to get DC (Darling Child) to stay in that position is to hold them … Is it ok for DC to sleep like that? (P47)

This quote indicated that the parents endeavoured to keep their baby on their back, but described there being no way to ensure that their babies remained in this position. They subsequently sought reassurance that this was acceptable despite their knowledge of safe sleep guidance. Parents frequently expressed distress in their posts that they struggled to sleep because they worried about their children's sleeping position. Another parent reported that their infant moved onto their front during sleep: ‘he flips himself over the second I put him down in the cot and settles himself on his front. He'll be ok won't he? If I roll him to his back all he'll breaks loose’ (P17). This parent similarly sought reassurance about their infant's sleeping position. A third post also referred to concern about back sleeping, with the parent describing repeatedly waking because they were worried about the infant's sleeping position (P19). These illustrate how parents sought reassurance when their infants' natural sleeping positions differed from their understanding of the guidance.

#### 3.4.4 Sub‐Theme 3.4: Sleeping Set‐Up

Parents also sought advice regarding their babies' sleeping set‐up, ranging from blanket use to the mattress type they were sleeping on. One parent mentioned blanket use:

I bedshare with my 2 mo (month old) I use a blanket it's fleece and she'll wear a onesie and a sleep sack. I made sure last night to have it tucked under her bottom and I had it on me only … But last night I woke up to her making a noise and thank GOD she did because the cover was over her face. (P100)

Many posts reflected awareness of the importance of sleeping safely with blankets, but co‐sleeping blanket usage was a concern, as parents recognized it could be unsafe for infants, but that they could not sleep comfortably without one. A parent asked, ‘he sleeps on top of our blanket now, but when can he be partially under it?’ (P113). Parents also discussed the mattress type most suitable for their child: ‘I know babies are supposed to sleep on a firm mattress. But our mattress is quite soft. Is this an issue when co sleeping?’ (P34). This showed an awareness of safe sleep guidance regarding the mattress their baby was sleeping on. Another parent considered bedsharing: ‘Thinking of just getting rid of the crib and putting a mattress on the floor and sleeping with her there’ (P111). Posts that discussed co‐sleeping highlighted that choosing a suitable mattress was challenging as parents had softer mattresses than the type recommended for infant sleep.

### 3.5 Question Types and Content Analysis Results of Responder Comments on the Posts

Across the dataset, three broad question types were identified. Parents most frequently sought practical advice on infant sleep arrangements and safe sleep practices (*n* = 85, 72%), followed by requests for reassurance about existing practices (*n* = 50, 42.4%) and clarification when conflicting information or uncertainty existed (*n* = 23, 19.5%). Percentages do not sum to 100% because posts could contain more than one question type. Less frequently, questions related to infant development, sleep equipment or wider parenting issues. These findings suggest that online parenting communities were primarily used to resolve uncertainty surrounding the practical implementation of safe sleep recommendations.

Most posts received responses from the other parents. A total of 525 responses to posts were analysed. The responses to the posts were analysed for the theme of the response, meaning code and type of response. Response types included shared experience, advice‐seeking, offering solutions, product‐related (e.g., recommendations), solidarity (e.g., normalization) and empathic. In terms of the types of responses, Figure 3 and the associated data table below offer a summary of frequencies and percentages for various responder themes identified in the data.

**FIGURE 3:**
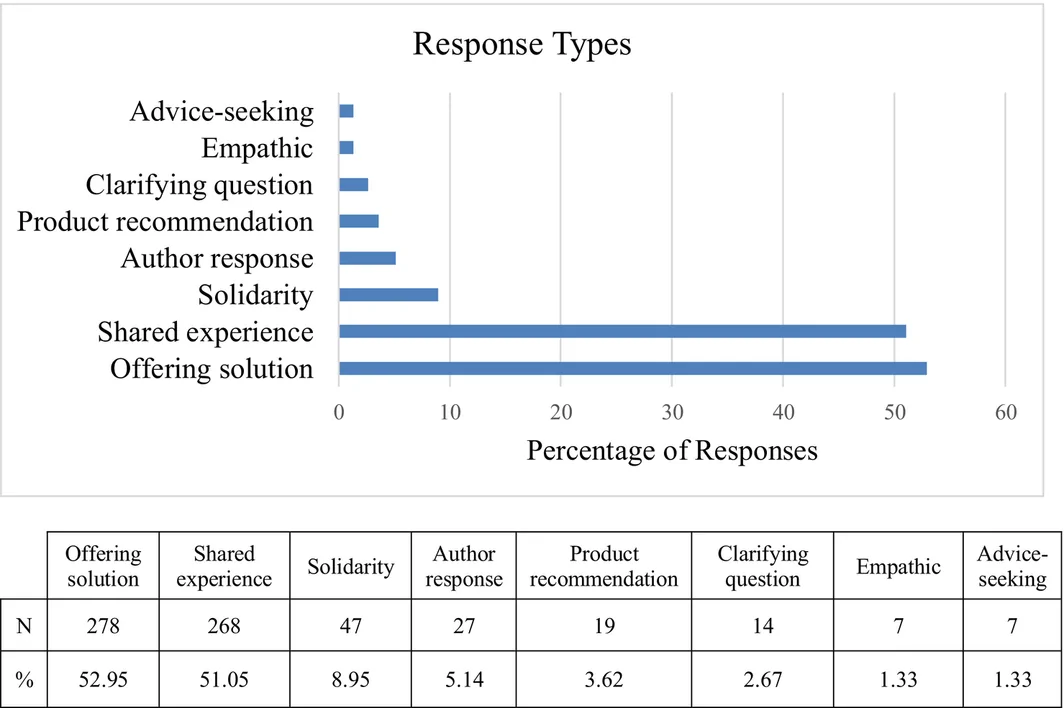
Predominance of response type. Horizontal bar chart showing percentages of response types in social media discussions about infant sleep. *Shared experience* and *offering solutions* were the most common responses, followed by *solidarity*, *author response* and *product recommendation*. *Advice‐seeking*, *empathic* and *clarifying question responses* were least frequent. An accompanying table shows the predominance of response types in social media discussions about infant sleep. The most frequent were *offering solutions* (52.95%) and *shared experiences* (51.05%), followed by *solidarity* (8.95%), *author response* (5.14%), *product recommendations* (3.62%), *clarifying questions* (2.67%) and *empathic* or *advice‐seeking responses* (both 1.33%).

Figure 3 summarizes the predominance of response types across the dataset. Offering solutions (52.9%, *n* = 278) and sharing personal experiences (51.1%, *n* = 268) were the dominant forms of peer interaction, indicating that responses were primarily characterized by the exchange of practical advice and lived experience. Expressions of solidarity (9.0%, *n* = 47) occurred less frequently, while author responses (5.1%, *n* = 27), product recommendations (3.6%, *n* = 19) and clarifying questions (2.7%, *n* = 14) represented a relatively small proportion of interactions. Empathic responses and advice‐seeking were the least common response types (both 1.3%, *n* = 7). Collectively, these findings suggest that parents predominantly responded by sharing experiential knowledge and offering practical solutions rather than providing emotional support alone. As individual responses frequently fulfilled more than one communicative function, percentages exceed 100%. These findings indicate that peer interactions were predominantly solution focused, with parents drawing on their own experiences to provide practical guidance, while emotional support played a comparatively smaller role.

Most of these responses were a mix of parents who shared their experiences with anecdotal challenges and outcomes. Parents shared what solutions worked or did not work for them and made suggestions based on this. Parents' responses were often very supportive, providing solidarity in difficult times and stressing that they would get through the hard times and that it would eventually work out. The comments normalized experiences and appeared valuable to the post's author when they responded (as demonstrated in the quote excerpts from the post and associated responses below).

POST (P626) I feel more comfortable when she's right next to me 24/7 (I have pretty bad postpartum anxiety), But I know it can be dangerous. I had a nephew that passed from SIDS (Sudden Infant Death Syndrome), and when I read about bed‐sharing it's heavily shamed and that's all they talk about is SIDS. But Then I found this group, and kind of felt relief, but still very apprehensive and nervous, So have you guys had your kiddos sleep next to you at night since they were born? I just need some kind of reassurance.

RESPONDENT (P626r1) I feel you. My kiddo could not be set down at all.

RESPONDENT (P626r2) Many professionals advocate safe bedsharing, popular magazines and media are all for it and there are a lot of good resources about it.

RESPONDENT (P626r3) My baby girl has been sleeping next to me since 2 days old. I'm no expert, but feel free to DM (direct message) me if you have any questions.

RESPONDENT (P626r4) I gave birth in a European country, where Healthcare staff is generally supportive of bedsharing. The midwife who helped me deliver at the hospital told me to put the baby in the bed with me. She slept with me since day 1 and all went well.

RESPONDENT (P626r5) This calculator might help you contextualize your risk. If you are breastfeeding and following best practice on bedsharing the likelihood is pretty low.

RESPONDENT (P626r6) I remember being in your position desperately looking for safe ways to get sleep because baby simply would not sleep.

RESPONDENT (P626r7) The countries where bed sharing is the norm have the lowest rates of SIDS. In my culture bed sharing is normal. I have slept with all 5 of my babies since birth. They're all alive.

Most of the time (94.86%), authors did not respond to any of the replies on their post. Solidarity and normalization were often intended to dilute parental guilt or societal judgement regarding safe sleep practices:

You are behaving normally and naturally honestly please understand I mean this in the kindest way possible … but you have got to develop a thick skin and learn to ignore … everyone will have an opinion on the way you mother your child. All that matters is you and your baby are happy with how you do things. (P19r1)

Responses on all three platforms included accounts of conflicting guidance from professional and peer sources, with respondents frequently drawing on their own experiences when discussing infant sleep practices.

The findings reflect that discussions across online parenting communities focused on practical aspects of infant sleep, as addressed in *Safer Sleep for Babies: A Guide for Parents* (The Lullaby Trust 2024), particularly sleeping conditions and sleeping arrangements. Parents most commonly sought practical advice and reassurance on implementing these recommendations, while responses were dominated by practical solutions and shared personal experiences. These patterns indicate that safe sleep guidance was represented alongside parents' experiential knowledge and practical accounts of infant sleep, with discussions reflecting how recommendations were considered in relation to everyday parenting experiences.

## 4 Discussion

The present study found that discussions across online parenting platforms were dominated by practical aspects of the infant sleep environment, while responses were characterized predominantly by the exchange of practical advice and personal experiences as demonstrated by the thematic prevalence analysis (Figure 2) and response‐type analysis (Figure 3). These findings suggest that parents use online communities not only to seek information about infant safe sleep but also to discuss and make sense of safe sleep recommendations within the context of everyday parenting.

This study explored how infant sleep practices were discussed across three online parenting platforms. To address the primary aim of this study by inductively understanding which aspects and themes of infant sleep were represented and negotiated across social media platforms Reddit and on parenting forum sites Netmums and Mumsnet, 119 posts were analysed alongside 525 corresponding responses. Posts covered a broad variety of aspects related to safe sleep practices and challenges, including sleep position, sleep surfaces, sleep safety products, room temperature and bed‐sharing or co‐sleeping. The proportion of posts on the different aspects of infant safe sleep was examined in a thematic prevalence analysis (Figure 2), which identified that sleeping conditions reflected the highest post prevalence. At the sub‐theme level, sleeping set‐up was the most prominent, followed by the competing needs of parents versus their babies. Most posts primarily focused on seeking support or advice. They often expressed feelings of emotional distress, confusion or difficulties in dealing with conflicting information from online sources or from healthcare professionals. Responses to posts were predominantly characterized by practical guidance and shared experiences, while expressions of normalization and solidarity were also present and sometimes expressed intentions to reduce feelings of guilt or criticism regarding safe sleep practices.

The secondary aim was to explore how parents represented and discussed aspects of infant safe sleep. The findings indicate that discussions were predominantly centred on practical aspects of the infant sleep environment, with parents frequently seeking and offering peer‐based advice when discussing how safe sleep recommendations might be applied in everyday contexts. The predominance of anecdotal responses and limited references to official guidance indicate that lived experience was a prominent feature of these discussions, sometimes alongside references to formal recommendations, particularly when parents described emotionally charged or uncertain decisions. These findings can be considered alongside concerns raised by Pease et al. (2017, 2021) regarding parental decision‐making around infant sleep. Consistent with the response‐type prevalence analysis (Figure 3), offering practical solutions and sharing personal experiences were the predominant response types, while expressions of solidarity provided additional emotional reassurance. These findings suggest that parents used online communities not only to obtain factual information but also to seek reassurance, validate decisions and contextualize discussions of safe sleep guidance within the realities of everyday parenting. The predominance of anecdotal responses and the limited references to official guidance further indicate that lived experience was a prominent source of information within these discussions, alongside relatively limited reference to formal recommendations.

To our knowledge, this is the first study to analyse the content of social media and parent forum posts regarding infant safe sleep practices in detail. In interpreting the findings in context, this study was undertaken in 2022, during a period in which digital technology was increasingly used as a source of information and psychosocial support.

The findings align with Paoletti et al. (2024) in illustrating how social media platforms function as spaces where parents seek reassurance, compare experiences and construct meaning around infant sleep practices. Rather than examining behavioural influence, this study describes the discourse through which parents discuss guidance and negotiate everyday challenges. Cole et al. (2022) conducted a scoping review that explored caregivers' awareness of safe sleep recommendations and identified practical challenges, cultural beliefs, conflicting advice and personal experiences as factors preventing compliance. Considering the findings of Cole et al. (2022) alongside this research analysis and noting the predominance of experience‐based response styles (vs. guideline based or healthcare based) raises concerns about the accuracy and application of the safe sleep guidance that caregivers receive.

Peer validation emerged as a recurrent discursive feature within these platforms. Online forums and social media provide spaces where parents exchange experiences, articulate concerns and calibrate their interpretations of infant care guidance. Prior research indicates that digital communities can support public health recommendations or circulate contradictory information depending on the nature of the discourse (Thoren et al. 2013; Kanchan and Gaidhane 2023; Bennett et al. 2024; Francisco 2024). The present study contributes to this literature by mapping how such discourse unfolds rather than examining behavioural outcomes.

The findings offer insight into how parents engage with infant safe sleep guidance within informal, peer‐led digital spaces. Rather than assessing behavioural influence, this study maps the discursive environment in which parents seek reassurance, negotiate conflicting advice and share lived experiences. The predominance of anecdotal responses suggests that peer‐generated narratives often hold greater conversational weight than formal guidance within these forums. This highlights a potential gap between lived parenting realities and the framing of official recommendations, underscoring the importance of understanding how safe sleep guidance is represented, discussed and contextualized in online communities.

The analysis of information sources (Table 1) further supports these findings. References to peer advice and general internet searches occurred more frequently than references to official safe sleep guidance, suggesting that parents often relied on informal, easily accessible sources when interpreting infant sleep recommendations. While this does not necessarily indicate a lack of trust in professional guidance, it highlights the prominence of informal digital sources within these discussions. These findings suggest that public health organizations may benefit from strengthening their presence within digital environments where parents actively seek advice, thereby improving the accessibility and visibility of evidence‐based safe sleep information.

Digital platforms serve as important sites of meaning‐making for parents, where emotional support, social comparison and shared experience are features of the discourse surrounding infant sleep. While previous research has examined how online information may affect parental decision‐making, the present study focuses specifically on the content and nature of these discussions. By analysing discourse rather than behaviour, this study contributes a nuanced understanding of how parents articulate concerns, seek validation and navigate guidance in peer‐led environments.

These findings highlight the value of engaging with parent‐led forums to better understand how safe sleep guidance is represented and discussed. Strengthening digital health communication may require bridging the gap between formal recommendations and the lived contexts parents describe in online conversations. By recognizing the discursive role of peer support networks, public health messaging can be better aligned with the realities parents face, fostering trust and improving the clarity and applicability of safe sleep guidance.

### 4.1 Limitations and Strengths

This study enhanced the analysis through investigator triangulation, with three researchers independently contributing to the coding process and refining the coding through collaborative discussion until consensus was reached. Although this approach strengthened the consistency and credibility of the analysis, qualitative interpretation is inherently subjective, and the potential for interpretive bias cannot be eliminated.

This snapshot of the observation and analysis of social media posts related to safe sleep practices was limited to a 6‐month sampling period across three parenting platforms. Furthermore, data extraction was limited to the original post and the first 10 direct responses to ensure a consistent and manageable approach across discussion threads of varying lengths. While this strategy provided sufficient information power to address the study aims, later comments may have contained additional perspectives that were not captured. The unequal distribution of eligible posts across platforms, particularly the relatively small number of Netmums discussions, also means that the findings may be more reflective of discussion patterns on Mumsnet and Reddit, particularly Mumsnet, than across online parenting communities more broadly.

Therefore, conducting longitudinal monitoring across a wider range of online parenting communities would provide a broader basis for investigating changes in infant sleep discussions and informing future targeted interventions. A limitation of the sampling process is that, because searches were conducted manually across platforms with differing search functions, a complete numerical record of all posts initially screened was not retained. Consequently, the number of posts considered before application of the eligibility criteria cannot be reported. The final dataset comprised posts that met the predefined eligibility criteria during the specified sampling period.

One strength of this study is that the data were extracted from naturally occurring social media posts, rather than from a controlled research setting. As such, the accounts are not shaped by researcher presence or questioning and may therefore reflect more candid, ecologically valid expressions of parents' experiences. The use of naturally occurring online discussions provided ecological validity but also presented ethical considerations associated with the use of publicly available data. These considerations were addressed through the ethical approach described in Section 2.

### 4.2 Implications for Practice, Policy and Further Research

These findings have implications for applied practice, policy and future research. Adopting a constructivist approach that recognizes that knowledge is co‐constructed through peer support in digital communities is a worthwhile focus for future research. Infant safety sleep guideline compliance is imperative to reduce the risk of SUID. However, parents in the present study frequently described experiencing existing safe sleep recommendations as difficult to implement within the realities of everyday parenting, particularly when balancing infant safety with competing practical demands.

The findings in this study are consistent with the findings of Altfeld et al. (2017) and Kruse et al. (2024) that parent–child co‐sleeping is often stigmatized because of discouraging attitudes from healthcare professionals, which can inhibit parental disclosure and engagement, whereas supportive socio‐cultural and familial attitudes tend to reinforce co‐sleeping practices. Our findings align with previous research suggesting that some parents perceive discussions around co‐sleeping as stigmatizing and may therefore be reluctant to disclose their sleep practices to healthcare professionals (Altfeld et al. 2017; Kruse et al. 2024). While debate continues regarding approaches to communicating bed‐sharing risks within the wider literature (Huber et al. 2024; Moon, Carlin and Hand 2022; Tappin et al. 2023), our findings suggest that parents value practical, non‐judgemental guidance that acknowledges the realities of infant care. These findings highlight the potential value of public health messaging that clearly communicates both evidence‐based risks and practical harm‐reduction strategies where appropriate. They also suggest that future policy development and dissemination strategies may benefit from considering how safe sleep guidance can be communicated in ways that support parents from diverse backgrounds and circumstances while maintaining evidence‐based safety recommendations.

The findings from this study indicate that while the best practice guidelines provide important safety recommendations, the parents in this study often experienced them as too inflexible for daily parenting practice. Given the developmentally dynamic and complex transitions through different infant and toddler sleep routines that parents navigate, some parents described finding it difficult to reconcile general recommendations with the practical realities of infant care, particularly as sleep routines evolved over time. Incorporating parents' experiences into guideline design and recognizing that they may at times need to adapt recommendations safely could offer alternative strategies or nuances that can empower parents to make informed decisions while still maintaining safe sleep practices. Therefore, co‐creating guidelines with inputs from parents and practitioners could increase practical applicability, reduce parental anxiety, normalize typical variations in infant sleep and ultimately support safer and more sustainable sleep routines. The findings suggest that future public health messaging may benefit from recognizing the practical challenges parents described when implementing safe sleep recommendations.

These findings highlight the need for social support for new parents and reliance on peers for reassurance and guidance. With the increasing impact of online peer support on parents of infants, understanding how safe sleep practices are represented on social media may inform more effective digital health communication policies and dissemination strategies. Various advanced electronic text mining tools have been developed that can advance the large‐scale monitoring of health education and awareness trends in social media data (Westrupp et al. 2022; Santos‐Pereira et al. 2022), which may be used to identify areas of misinformation. Given their widespread popularity, additional research on parenting platforms is needed to comprehend the motivations, consequences and risks associated with dynamic online communication among parents seeking guidance on infant sleep practice.

Therefore, it is imperative to explore the impact of parents' social media use throughout their lives and their respective children's lives in future investigations into infant care practices and highlight the value of collaborating with parent‐led forums to promote best practice guidelines, tackle misinformation and foster trust in health communication. There are implications for improving public health messaging and digital intervention. This study emphasizes how safe sleep practices are discussed in online communities. Therefore, health psychologists and public health agencies should consider the impacts of these platforms when developing individual‐ and societal‐level interventions. These findings support informing digital health initiatives that promote safe sleep practices by fostering trust and considering the realities that parents face.

## Author Contributions

**Kirsty L. Hodgson:** writing – original draft, visualization, writing – review and editing, formal analysis, methodology, investigation. **Kayleigh J. Ison:** investigation, writing – original draft, writing – review and editing, formal analysis. **Madison S. Sharp:** investigation, writing – review and editing, formal analysis. **Sarah Snuggs:** conceptualization, investigation, funding acquisition, writing – original draft, methodology, validation, visualization, writing – review and editing, formal analysis, supervision, project administration. **Davina Ledermann:** conceptualization, investigation, writing – review and editing, methodology. **Natalie A. Masento:** conceptualization, investigation, writing – review and editing, methodology. **Katie L. Barfoot:** conceptualization, investigation, funding acquisition, writing – original draft, methodology, validation, visualization, writing – review and editing, formal analysis, project administration, supervision.

## Funding

Funding was received from the University of Reading Undergraduate Research Opportunities Placement (UROP) scheme in the summer of 2022. The UROP project funding enabled undergraduate students to support mentored research projects for researchers in the Psychology Department. The funders of this research had no role in the study design, conduct, data collection, analysis, manuscript preparation or submission.

## Ethics Statement

The study was conducted in accordance with the Declaration of Helsinki and was approved by an Institutional Review Board/Ethics committee. See details under Section 2.

## Consent

This study utilized secondary data that had already been published online, it was not possible to obtain informed consent from all users of each social media group. To overcome this, a post was published on the main page of the group by the researcher to all members that explained the aims and rationale of the research, data protection information and assurances of the anonymization of identifying information during extraction and analysis. Details were also provided to inform individuals on how to ‘dissent consent’ (not consent) and withdraw their data from use in the research. This research did not require proactive input from participants, unless they chose to withdraw their data from inclusion in the study by contacting the researcher at any time. None of the participants expressed concerns or requested data extraction throughout or after this study. This follows guidance from the Information Commissioner's Office, which stipulates that if personal data are obtained from publicly accessible sources (such as social media), individuals must be provided with private information (Information Commissioner's Office 2023).

## Conflicts of Interest

The authors declare no conflicts of interest.

## Supporting information


**Supplementary File 1** Search strategy and keyword framework.

**Supplementary File 2** Coding structure and analytic process (illustrative examples).

## Data Availability

Data are available upon reasonable request from the authors.
