# Supplementary material for: Sleep, Safety and Social Media: A Qualitative Analysis of Online Discussions About Infant Sleep
Source: Child Care Health Dev. 2026 Nov 4;52(6):e70371. doi: 10.1111/cch.70371 (PMC13734727; doi:10.1111/cch.70371)
Supplement: Supplementary file 1 [file CCH-52-e70371-s001.docx]

**Supplementary File 1. Search Strategy and Keyword Framework**

**Development of the search strategy**

The search strategy was developed *a priori* using contemporary evidence-based recommendations for infant safe sleep published by the American Academy of Pediatrics (AAP), the National Institute for Health and Care Excellence (NICE), The Lullaby Trust, and the Basis Baby Sleep Information Source (Basis) (Moon et al., 2022; National Institute for Health and Care Excellence [NICE], 2021; The Lullaby Trust, 2024).

The Lullaby Trust guidance included recommendations relating to the infant sleep environment, including use of a firm, flat mattress; avoidance of pillows, quilts, duvets, bumpers, weighted bedding, pods, nests and sleep positioners; keeping the infant's head uncovered and avoiding overheating; placing U infant at the bottom of the cot; maintaining a clear sleep space; room-sharing during the first six months; and avoiding routine sleep in bouncy chairs, beanbags, car seats and other seated positions (The Lullaby Trust, 2024).

The search strategy was informed by these recommendations to maximise the likelihood of identifying discussions relating to infant sleep and recognised safe sleep practices. As parents may not explicitly use clinical terminology when discussing infant sleep online, both professional terminology and commonly used parental language were incorporated. Search terms therefore included medical terminology (e.g., supine, Sudden Infant Unexpected Death), commonly used abbreviations (e.g., SIDS, SUID), spelling variations (e.g., bedsharing, bed sharing, co-sleep, cosleep), and colloquial terms frequently used within parenting communities.

Searches were undertaken manually within each platform according to the platform-specific procedures described in the Methods. Where platform search functions were limited, posts were identified through manual screening of discussions containing one or more of the search terms listed in Table S1. Singular and plural forms, spelling variations and synonymous terms were considered during data extraction to maximise sensitivity while maintaining consistency across platforms. Posts were screened sequentially until eligible discussions within the predefined sampling period had been identified, resulting in a final dataset of 118 posts.

The keyword framework was designed to maximise search sensitivity by combining evidence-based terminology with commonly used parental language, abbreviations and spelling variants. It was used solely to identify potentially relevant discussions for inclusion and did not constitute a deductive coding framework for the subsequent qualitative analysis.

**Table S1.**

*Keyword framework used for data extraction*

| Safe sleep domain | Keywords and search terms |
| --- | --- |
| **General infant sleep** | sleep, asleep, sleeping, bedtime, bed time, bed, nap, naps, napping, night, nighttime, night time, overnight |
| **Safe sleep guidance** | safe sleep, safer sleep, safe sleeping, sleep safety, guidance, guidelines, recommendations, advice |
| **Sleep-related infant deaths** | Sudden Infant Death Syndrome, SIDS, Sudden Unexpected Infant Death, SUID, Sudden Unexpected Death in Infancy, SUDI, cot death, crib death, infant death |
| **Infant sleep position** | back sleeping, on back, on their back, supine, tummy sleeping, on tummy, front sleeping, prone, side sleeping, side position, positioning |
| **Feet-to-foot positioning** | feet to foot, feet-to-foot, bottom of cot, foot of cot |
| **Flat sleep surface** | flat, firm mattress, flat surface, sleep positioner, positioner, wedge, reflux wedge, sleep wedge, pod, nest, infant nest |
| **Clear sleep space** | blanket, blankets, muslin, comforter, comfort toy, toy, teddy, pillow, pillows, cushion, quilt, duvet, cot bumper, bumper |
| **Sleep environment temperature** | room temperature, thermometer, degrees, Celsius, Fahrenheit, overheating, hot, heat, cold, freezing, clothing, layers, sleeping bag, sleep sack, Grobag |
| **Infant sleep location** | cot, crib, Moses basket, bassinet, bedside crib, bedside sleeper, baby box |
| **Alternative sleep locations** | car seat, infant carrier, bouncer, bouncy chair, rocker, swing, pram, pushchair, buggy, stroller, sofa, couch, settee, armchair |
| **Bed-sharing and co-sleeping** | bed sharing, bedsharing, share bed, co-sleeping, co sleeping, co-sleeping, family bed |
| **Bed-sharing risk factors** | alcohol, drinking, drunk, tipsy, smoking, smoker, cigarette, tobacco, vape, vaping, drugs, medication, pills, sedatives |
| **Infant vulnerability** | premature, preterm, preemie, low birth weight, LBW |
| **Sleep environment hazards** | trapped, wedged, entrapment, wall and mattress, mattress gap, roll out, fall out, bed rail |
| **Others sharing the sleep surface** | sibling, brother, sister, toddler, other child, son, daughter, pet, cat, dog |
| **Infant feeding and sleep** | breastfeeding, breast feeding, nursing to sleep, feeding to sleep, bottle feeding, overnight feeding |

**Development of the keyword framework from contemporary safe sleep guidance**

The keyword framework was informed by principal recommendations reported across UK and international safe sleep guidance (Moon et al., 2022; NICE, 2021; The Lullaby Trust, 2024). Specifically, search terms reflected recommendations relating to:

- placing infants supine (on their back) for every sleep;
- using a firm, flat sleep surface;
- maintaining a clear sleep environment free from loose bedding, pillows and soft objects;
- ensuring infants sleep in an appropriate sleep space, such as a cot, crib or Moses basket;
- avoiding routine sleep in car seats, sofas, armchairs and other sitting devices;
- recognising circumstances associated with increased risk during bed-sharing, including parental smoking, alcohol consumption, drug use and infant prematurity;
- maintaining an appropriate sleep environment temperature and avoiding overheating; and
- promoting safer sleep through evidence-based infant care practices.

The inclusion of broader sleep-related terminology (e.g., sleep, bedtime, nap) ensured that discussions potentially relevant to infant safe sleep were captured even when users did not explicitly reference recognised safe sleep terminology. This approach prioritised search sensitivity while maintaining transparency and reproducibility across the three platforms.

Importantly, the keyword framework was used to identify potentially relevant discussions for inclusion and was not applied as a deductive coding framework during qualitative analysis. The subsequent thematic analysis was inductive and data-led, with safe sleep guidance used only to contextualise and interpret findings relating to infant safe sleep practices.

**References**

Basis Baby Sleep Information Source. (2024). *Safer sleep for babies*. Durham University. <https://www.basisonline.org.uk/>

Blair, P. S., Sidebotham, P., Pease, A., & Fleming, P. J. (2014). Bed-sharing in the absence of hazardous circumstances: Is there a risk of sudden infant death syndrome? *PLoS ONE, 9*(9), e107799. <https://doi.org/10.1371/journal.pone.0107799>

Carpenter, R., McGarvey, C., Mitchell, E. A., Tappin, D. M., Vennemann, M. M., Smuk, M., & Carpenter, J. R. (2013). Bed sharing when parents do not smoke: Is there a risk of sudden infant death syndrome? An individual level analysis of five major case-control studies. *BMJ Open, 3*, e002299. <https://doi.org/10.1136/bmjopen-2012-002299>

Moon, R. Y., Carlin, R. F., & Hand, I. (2022). Sleep-related infant deaths: Updated 2022 recommendations for reducing infant deaths in the sleep environment. *Pediatrics, 150*(1), e2022057990. <https://doi.org/10.1542/peds.2022-057990>

National Institute for Health and Care Excellence. (2021). *Postnatal care* (NG194). <https://www.nice.org.uk/guidance/ng194>

The Lullaby Trust. (2024). *Safer sleep advice*. <https://www.lullabytrust.org.uk/safer-sleep-advice/>
