# Supplementary material for: Sleep, Safety and Social Media: A Qualitative Analysis of Online Discussions About Infant Sleep
Source: Child Care Health Dev. 2026 Nov 4;52(6):e70371. doi: 10.1111/cch.70371 (PMC13734727; doi:10.1111/cch.70371)
Supplement: Supplementary file 2 [file CCH-52-e70371-s002.docx]

**Supplementary File 2. Coding Structure and Analytic Process (illustrative examples)**

**Development of the coding structure**

Coding was undertaken iteratively and was primarily inductive and data-led. Initial coding involved identifying the substantive topic or issue represented within each post or response. Initial codes were then compared across posts and platforms to identify recurring meanings and relationships. Codes were not fixed at the outset; they were added, combined, refined or renamed when comparison across the dataset indicated that the initial coding did not adequately capture recurring meanings or distinctions within the data. Conceptually similar codes were consolidated or grouped into higher-order categories, which were subsequently refined into subthemes and overarching themes through iterative discussion and consensus between the three researchers. The safe sleep guidance was not used as a deductive coding framework. Rather, it informed the development of the search strategy and was subsequently used to contextualise and interpret findings relating to infant safe sleep practices.

***Illustrative examples of the coding process drawn from the data workbook***

| **Raw data/extract** | **Initial Code** | **Meaning Code** | **Subtheme** | **Overarching Theme** |
| --- | --- | --- | --- | --- |
| *"I currently co-sleep with my 7 week old... I'm looking for advice on how to end it..."* | Difficulty transitioning from co-sleeping | Parent versus baby's needs | Parent versus baby's needs | Competing priorities |
| *"I have adhered to all of the Lullaby Trust advice for safe sleep..."* | Following guidance while managing developmental change | Following safe sleep guidelines | Following safe sleep guidelines | Competing priorities |
| *"I don't want to co sleep... but... every hour rather than every half hour..."* | Co-sleeping as a response to repeated waking | Balancing parental and infant needs | Parent versus baby's needs | Competing priorities |

The examples above illustrate the progression from raw textual data to initial codes, meaning codes, subthemes and overarching themes. Initial codes described the substantive content of an extract, whereas meaning codes captured the broader interpretive meaning represented across related extracts. Conceptually similar codes were compared across the dataset and, where appropriate, consolidated or grouped into higher-order categories. These categories were subsequently refined into subthemes and overarching themes through review, discussion and consensus between the researchers. The examples represent the final coding structure developed through the iterative analysis rather than an *a priori* framework imposed on the dataset.
